# Oxidative ornithine metabolism supports non-inflammatory *C.* *difficile* colonization

**DOI:** 10.1038/s42255-021-00506-4

**Published:** 2022-01-06

**Authors:** Kali M. Pruss, Fatima Enam, Eric Battaglioli, Mary DeFeo, Oscar R. Diaz, Steven K. Higginbottom, Curt R. Fischer, Andrew J. Hryckowian, William Van Treuren, Dylan Dodd, Purna Kashyap, Justin L. Sonnenburg

**Affiliations:** 1 Department of Microbiology and Immunology, Stanford University School of Medicine, Stanford, CA USA; 2 Department of Medicine, Division of Gastroenterology and Hepatology, Mayo Clinic, Rochester, MN USA; 3 ChEM-H, Stanford University, Stanford, CA USA; 4 Department of Pathology, Stanford University School of Medicine, Stanford, CA USA; 5 Department of Physiology and Biomedical Engineering, Mayo Clinic, Rochester, MN USA; 6 Center for Human Microbiome Studies, Stanford, CA USA; 7 Present Address: Department of Molecular and Cellular Biology, Kennesaw State University, Kennesaw, GA USA; 8 Present Address: Octant Bio, Emeryville, CA USA; 9 Present Address: Department of Medicine, Division of Gastroenterology and Hepatology, University of Wisconsin School of Medicine and Public Health, Madison, WI USA; 10 Present Address: Department of Medical Microbiology and Immunology, University of Wisconsin School of Medicine and Public Health, Madison, WI USA

**Keywords:** Gene expression analysis, Bacterial pathogenesis, Microbial genetics, Metabolism, Clostridium difficile

## Abstract

The enteric pathogen *Clostridioides* *difficile* (*Cd*) is responsible for a toxin-mediated infection that causes more than 200,000 recorded hospitalizations and 13,000 deaths in the United States every year^1^. However, *Cd* can colonize the gut in the absence of disease symptoms. Prevalence of asymptomatic colonization by toxigenic *Cd* in healthy populations is high; asymptomatic carriers are at increased risk of infection compared to noncolonized individuals and may be a reservoir for transmission of *Cd* infection^2,3^. Elucidating the molecular mechanisms by which *Cd* persists in the absence of disease is necessary for understanding pathogenesis and developing refined therapeutic strategies. Here, we show with gut microbiome metatranscriptomic analysis that mice recalcitrant to *Cd* infection and inflammation exhibit increased community-wide expression of arginine and ornithine metabolic pathways. To query *Cd* metabolism specifically, we leverage RNA sequencing in gnotobiotic mice infected with two wild-type strains (630 and R20291) and isogenic toxin-deficient mutants of these strains to differentiate inflammation-dependent versus -independent transcriptional states. A single operon encoding oxidative ornithine degradation is consistently upregulated across non-toxigenic *Cd* strains. Combining untargeted and targeted metabolomics with bacterial and host genetics, we demonstrate that both diet- and host-derived sources of ornithine provide a competitive advantage to *Cd*, suggesting a mechanism for *Cd* persistence within a non-inflammatory, healthy gut.

## Abstract (web summary)

Using a combination of metabolomics and bacterial and host genetics, Pruss et al. show that upregulated oxidative ornithine metabolism in *Clostridioides* *difficile* promotes its persistence within the gastrointestinal tract under non-inflammatory conditions.

## Main

*Clostridioides* *difficile* (recently reclassified from *Clostridium*, here referred to as *Cd*) is the leading cause of nosocomial diarrhea worldwide. *Cd* causes disease by the secretion of large glycosylating toxin proteins^4^; in susceptible hosts, infection can result in toxic megacolon or death. An important facet of *Cd* biology is its persistence in humans in the absence of symptoms, termed asymptomatic carriage. Prevalence reports vary widely^2,5–9^; up to 71% of infants and 15% of healthy adults may asymptomatically carry toxigenic *Cd* strains, with estimates increasing in populations with underlying conditions^2^. Mechanisms underlying this aspect of *Cd* lifestyle remain obscure; an improved understanding of *Cd* metabolic behavior in non-inflammatory (homeostatic) versus inflammatory (generated by toxin production) conditions will better inform addressing and preventing disease progression.

Detailed investigations of *Cd* during asymptomatic carriage are challenging due to difficulties associated with (1) procuring sufficient human samples in the absence of diarrhea or disease, (2) the relatively low abundance of *Cd* in the human microbiota and (3) experimentally controlling toxin production in vivo. An approach we^10^ and others^11^ have taken to understand how toxin production in vivo alters *Cd* metabolism utilizes isogenic mutant strains of *Cd* with impaired toxin production; however, these studies focused on wild-type (WT) infection and attention toward *Cd* persistence in the absence of disease is warranted.

**Fig. 1:**
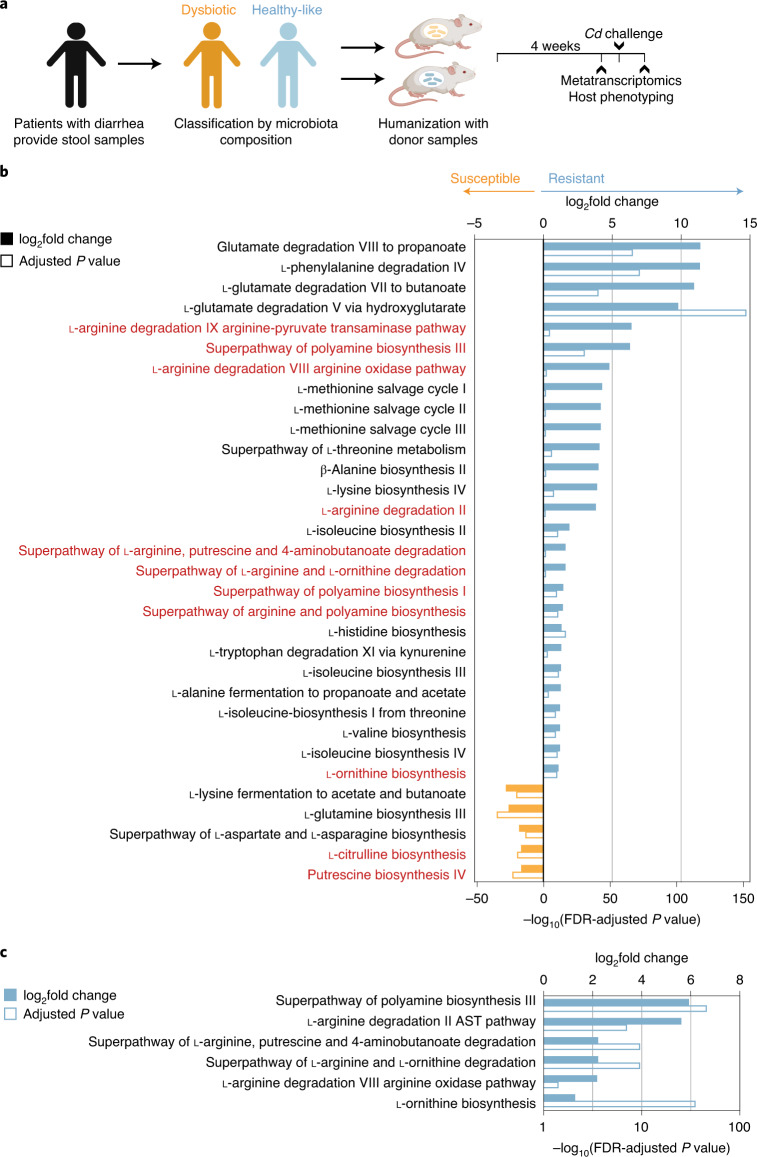
Community amino acid metabolic pathways differ in CDI-susceptible hosts. **a**, Experimental design. Samples from patients with diarrhea were collected, classified as healthy-like or dysbiotic and used for a humanized mouse model of CDI^13^. Mice humanized with healthy-like microbiotas are recalcitrant to developing CDI and associated pathology, whereas mice colonized with communities from dysbiotic, symptomatic patients harbor persistent infections and higher levels of inflammation. RNA-seq was performed on *n* = 6 mice per group. **b**, Community-level amino acid metabolic pathways enriched in mice humanized with healthy-like human microbiotas (positive log_2_fold change and log_10_adjusted *P* value) versus dysbiotic (negative). Metabolic pathways involved in ornithine, arginine and polyamine metabolism are colored in red. **c**, Differential expression of arginine, ornithine and polyamine metabolic pathways after *Cd* challenge. Source data

A previous study showed that humans colonized with naturally occurring non-toxigenic *Cd* isolates (*n* = 4) had distinct fecal metabolomes from those infected with toxigenic strains (*n* = 6)^12^, suggesting that microbial community metabolism was influenced by disease symptoms imparted by toxin production. To further understand changes to community metabolism during inflammatory versus non-inflammatory *Cd* infection (CDI), we examined metatranscriptomic data from a clinically relevant mouse model. Patients with diarrhea provided stool samples for microbiota sequencing; samples were classified as healthy-like or dysbiotic based on symptoms and microbial composition (Fig. 1a). Mice humanized with stool samples from healthy-like patients were recalcitrant to CDI, whereas mice humanized with feces from dysbiotic donors sustained high levels of *Cd* and higher inflammation scores^13^. Here, we compared expression of community-level pathways in cecal metatranscriptomes from these mice.

Hundreds of pathways were differentially expressed between dysbiotic (82 pathways enriched) and healthy-like (219 pathways) humanized mice. Of these, 28 pathways involved in amino acid metabolism were more highly expressed in healthy-like mice (13% of total differentially expressed), whereas only 6 amino acid metabolic pathways were enriched in dysbiotic mice (7%; Fig. 1b). The increase in expression of pathways involved in amino acid metabolism in healthy-like, *Cd*-resistant communities was accompanied by reduced levels of several amino acids in feces^13^. Several pathways involved in arginine, ornithine and polyamine metabolism were more highly expressed in healthy communities (Fig. 1b). These inter-related metabolic pathways were of interest as the metabolism of arginine and ornithine into polyamines enhances gut barrier function by increasing expression of tight junction proteins and lessening inflammatory responses in immune cells^14–16^. In a separate study that conducted metabolomic profiling of humans infected with *Cd* versus healthy controls, microbial amino acid metabolites were the strongest differentiators of *Cd* colonization; of these, ornithine had the second-highest odds ratio^17^. Furthermore, excess arginine and arginine di-peptides increase toxin production in some *Cd* strains in vitro^18^. Of the 11 arginine/ornithine/polyamine pathways enriched in healthy-like microbiotas before CDI, 6 remained more highly expressed after exposure to *Cd* (Fig. 1c). In dysbiotic mice, no pathways involved in arginine, ornithine nor polyamine metabolism were enriched after CDI (Fig. 1c). These results suggest the potential for community amino acid metabolism in enhancing host barrier function and playing a role in maintaining the gut in a non-inflammatory state when challenged with *Cd*. However, the role of *Cd* amino acid metabolism within recalcitrant versus susceptible communities remains a difficult question to address in the context of a complex microbiota.

**Fig. 2:**
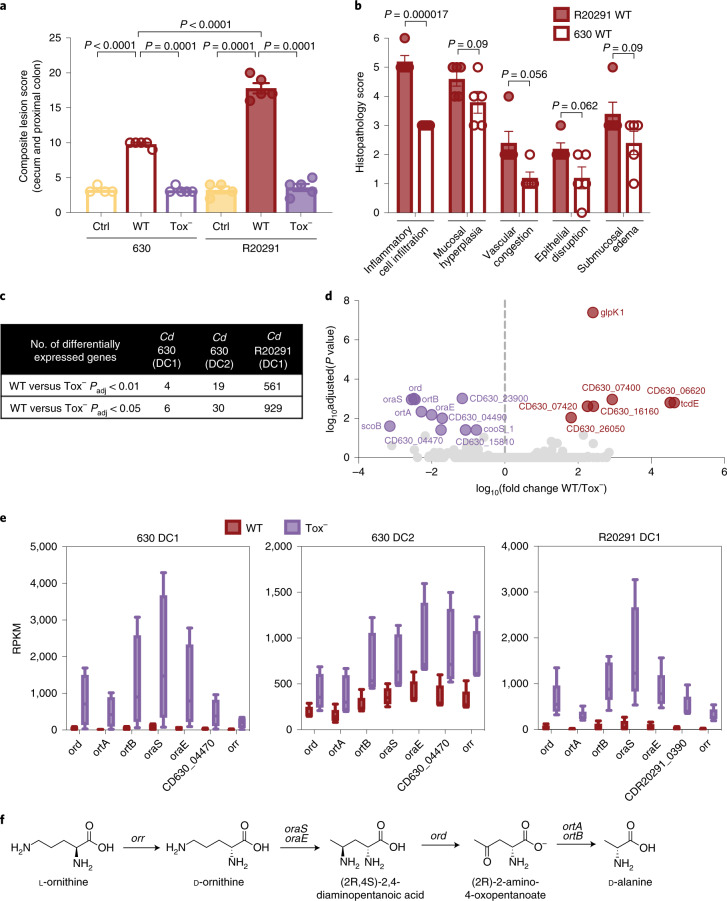
Putative l-ornithine oxidative degradation locus consistently differentially expressed in gnotobiotic models for *Cd* metabolism in a non-inflamed environment. **a**, Hypervirulent WT *Cd* R20291 elicits a significantly greater magnitude of pathology in the cecal blind tip and proximal colon of mice than all other groups, including WT *Cd* 630. Both WT infections (630 and R20291) incite significantly higher pathology than all Tox^−^ and uninfected control (crtl) groups, which do not differ from one another (one-way analysis of variance (ANOVA) F_(5,22)_ = 180.3 with Tukey’s post hoc comparisons. WT 630 versus R20291 Tox^−^ and control group, *P* < 0.0001; WT R20291 versus 630 Tox^−^ and control group, *P* < 0.0001; *n* = 5 mice per group except for the 630 uninfected control where *n* = 4; bars denote mean ± s.e.m.). **b**, Histopathological score breakdown for mice infected with WT R20291 or WT 630. R20291 leads to a significantly higher level of inflammatory cell infiltration (multiple unpaired Student’s *t*-tests with Benjamini, Krieger, Yekutieli two-stage linear step-up procedure; *n* = 5 mice per group; bars denote mean ± s.e.m.). **c**, Extent of differential gene expression between WT and Tox^−^ *Cd* across different gnotobiotic settings (DESeq2 Wald test). DC1 (*n* = 5 WT, *n* = 4 Tox^−^); DC2 (630, *n* = 4 mice per group; R20291, *n* = 5 mice per group). **d**, Combined differential gene expression between 630 WT (positive fold change) and 630 Tox^−^ (negative) combined across both defined communities (adjusted *P* value < 0.01, *n* = 9 WT, *n* = 8 Tox^−^). **e**, Normalized expression values (reads per kilobase million) of a putative l-ornithine degradation locus, the only genomic locus consistently differentially expressed across *Cd* strains and two different defined communities (box-plots denote interquartile range, whiskers denote min to max). **f**, Schematic of the ornithine oxidative degradation pathway; l-ornithine undergoes racemization to d-ornithine (*orr*, ornithine racemase), migration of the amino group from C5 to C4 (*oraSoraE*, d-ornithine aminomutase), a dehydrogenase reaction generating ammonia (*ord*, 2,4-diaminopentanoate dehydrogenase), CoA-mediated thiolytic cleavage generating acetyl-CoA (*ortAortB*, 2-amino-4-ketopentanoate thiolase) leading to ATP production via acetyl phosphate. Source data

**Extended Data Fig. 1:**
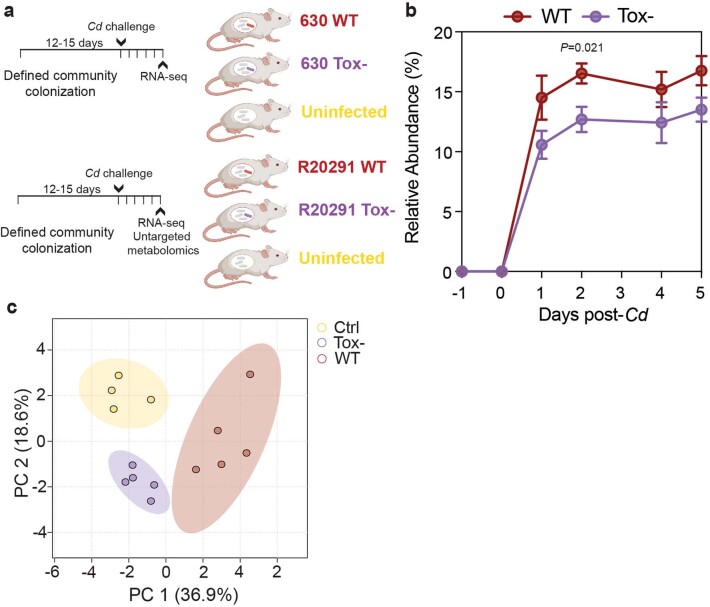
*Cd t*oxin production confers advantage *in vivo* and generates distinct metabolome. **a**, Experimental gnotobiotic mouse model for *Cd* colonization in non-inflamed conditions. Germ-free mice were tri-colonized with *B. thetaiotaomicron*, *C. sporogenes* and *E. coli* 12–15 days prior to infection with WT (630 or R20291) or Tox- (630 TcdA^−^TcdB^−^ or R20291 TcdA^−^TcdB^−^CDT^−^ *Cd* or gavaged with PBS. Mice were maintained on standard diet and no antibiotic pretreatment was used. **b**, WT R20291 reaches a higher relative abundance in the presence of a defined commensal community (DC 1, mean ± s.e.m., n = 5 mice/group, unpaired two-tailed t-test) than its toxin-deficient counterpart. Absolute abundance (CFU/mL feces) of the two strains did not differ (data not shown). **c**, R20291 toxin production differentiates the metabolic milieu of cecal contents. Principal components analysis with 95% confidence regions for mice infected with WT (n = 5 mice, red), Tox- (n = 5, purple) or uninfected controls (n = 4, yellow). Source data

To query changes to metabolism due specifically to *Cd*-induced inflammation versus the presence of *Cd* in a commensal (non-inflammatory) state, we employed toxin-possessing (WT) and toxin-deficient (Tox^−^) isogenic mutants of two *Cd* strains in defined communities of commensal microbes in gnotobiotic mice. Germ-free mice were colonized with a three-member community: *Bacteroides* *thetaiotaomicron*, *Escherichia* *coli* and *Clostridium* *sporogenes* (Defined Community 1, DC1), members of the three most abundant phyla in the adult human gut microbiota. After 12–15 d of colonization, mice were infected with either WT *Cd* (630 WT) or the toxin-deficient isogenic mutant (630 TcdA^−^B^−^, here referred to as 630 Tox^−^, which has insertional mutations in each of its genes encoding toxin proteins, *tcdA* and *tcdB*^19^; Extended Data Fig. 1a). WT 630 induced significantly more pathology than Tox^−^-colonized mice, which did not differ significantly from uninfected controls (Fig. 2a). We were interested in comparing *Cd* 630 with a hypervirulent strain, R20291, which produces a third toxin (binary toxin, CDT), increased levels of TcdA and TcdB^20^ and has been associated with more recent hospital outbreaks^21^. WT R20291 induced greater overall tissue damage (Fig. 2a) and more inflammatory cell infiltrates than 630 WT (Fig. 2b). Colonization with the isogenic triple-toxin knockout, which contains mutations in all three toxin genes (*tcdA*, *tcdB*, *cdt*; TcdA^−^B^−^CDT^−^ (ref. ^22^), here referred to as R20291 Tox^−^), led to an identical histopathology score as colonization with 630 Tox^−^ and both uninfected control groups (Fig. 2a). WT *Cd* reached a higher relative abundance than its toxin-deficient counterpart (Extended Data Fig. 1b), indicating that toxin production confers a competitive advantage to *Cd* in vivo.

**Extended Data Fig. 2:**
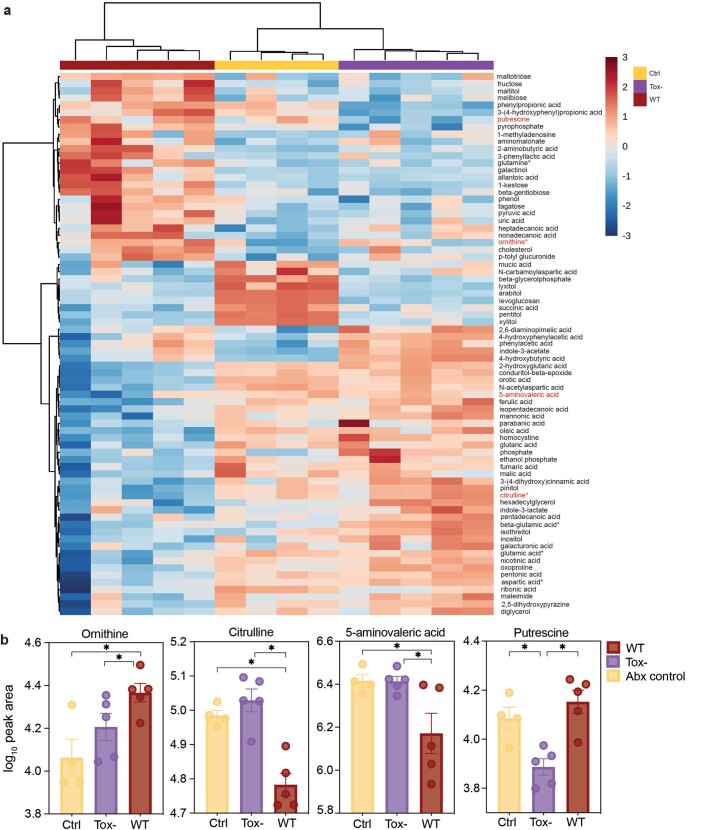
Untargeted metabolomics demonstrates toxin-dependent changes to metabolites in gut lumen. **a**, 75 metabolites with the greatest variance across groups of mice infected with R20291 WT (n = 5 mice), Tox- (n = 5) or uninfected controls (n = 4) as described in Extended Data Fig. 1a (row-normalized log_10-_transformed peak areas shown). Amino acids are noted with asterisks; metabolites in (**b**) are highlighted in red. **b**, Normalized peak values for identified metabolites related to ornithine metabolism. Ornithine and its reductive end-product 5-aminovaleric acid exhibit inverse trends (group sizes are as in **a**, mean ± s.e.m. shown; one-way ANOVA across all named metabolites with FDR-adjusted P value (*P*adj) < 0.1, * denotes significance by Fisher’s LSD post hoc comparison: ornithine F = 5.5103, *P*adj = 0.0849; citrulline F = 20.078, *P*adj = 0.0029, 5-aminovaleric acid F = 5.3369, *P*adj = 0.0895; putrescine F = 11.315, *P*adj = 0.0162). Source data

We performed untargeted metabolomics on cecal contents of mice infected with R20291 WT, R20291 Tox^−^ or uninfected controls. Identified metabolites of the gut lumen differentiate mice infected with toxin-producing *Cd* from Tox^−^ or the uninfected group (Extended Data Fig. 1c). Several amino acids and their bacterial metabolites were altered across infection conditions (Extended Data Fig. 2a and Supplementary Table 1). Notably, ornithine, putrescine (one product of microbial ornithine metabolism) and citrulline (a potential precursor) differed significantly across groups (Extended Data Fig. 2b). 5-Aminovalerate, the reductive product of ornithine and/or proline^17,23,24^, was higher in the absence of toxin-induced inflammation (Extended Data Fig. 2b). These metabolomics data combined with the community-level transcriptional pathway analysis in susceptible versus resistant humanized mice support the notion that metabolic pathways involving arginine, ornithine and putrescine are more active in the absence of *Cd*-induced inflammation.

To examine the metabolic behavior of *Cd* itself, we isolated RNA from cecal contents of mice 5 d after infection with WT (630 or R20291), Tox^−^ (630 or R20291) or uninfected controls for RNA-seq (Extended Data Fig. 1a). Using a defined commensal community allowed for sufficient coverage of the *Cd* transcriptional profile for differential expression analysis, which was not possible in a complex, humanized microbiota^13^. R20291, which elicited a greater degree of tissue damage (Fig. 2a) and increased inflammatory cell infiltrates (Fig. 2b), exhibited a far greater extent of differential gene expression due to toxin production than 630 (Fig. 2c and Supplementary Tables 2 and 3). We employed a second defined community (Defined Community 2, DC2), consisting of *Edwardsiella* *tarda*, *Clostridium* *scindens* and *Bacteroides* *thetaiotaomicron*, to determine whether the limited transcriptional response of *Cd* 630 was specific to the commensals of DC1. Again, few genes were differentially expressed by 630 in the presence or absence of toxin production (Fig. 2c). We combined *Cd* 630 gene expression across both defined communities to determine which genes were differentially expressed due to toxin production independent of the specific community members present (Fig. 2d and Supplementary Table 2). A gene annotated as a glycerol kinase (glpK1) was most highly enriched in WT, as well as two neighboring genes putatively annotated as a pyridoxal phosphate-dependent aminotransferase (CD630_07400) and an ethanolamine utilization protein (CD630_07420). WT R20291 also upregulated ethanolamine utilization (Supplementary Table 3), consistent with enteric pathogen ethanolamine metabolism in inflammatory conditions^25–27^.

In the absence of inflammation (Tox^−^), pathways for butyrate fermentation were more highly expressed in both hypervirulent R20291 (Supplementary Table 3) and 630 (DC2; Supplementary Table 2). Despite the smaller magnitude of differential expression in 630 compared to R20291 (Fig. 2c), a single transcriptional unit was differentially expressed across both commensal communities and was shared with R20291. A putative oxidative l-ornithine degradation pathway was more highly expressed by 630 Tox^−^ and R20291 Tox^−^ (Fig. 2e), suggesting that this pathway supports *Cd* metabolism in a non-inflamed gut.

**Extended Data Fig. 3:**
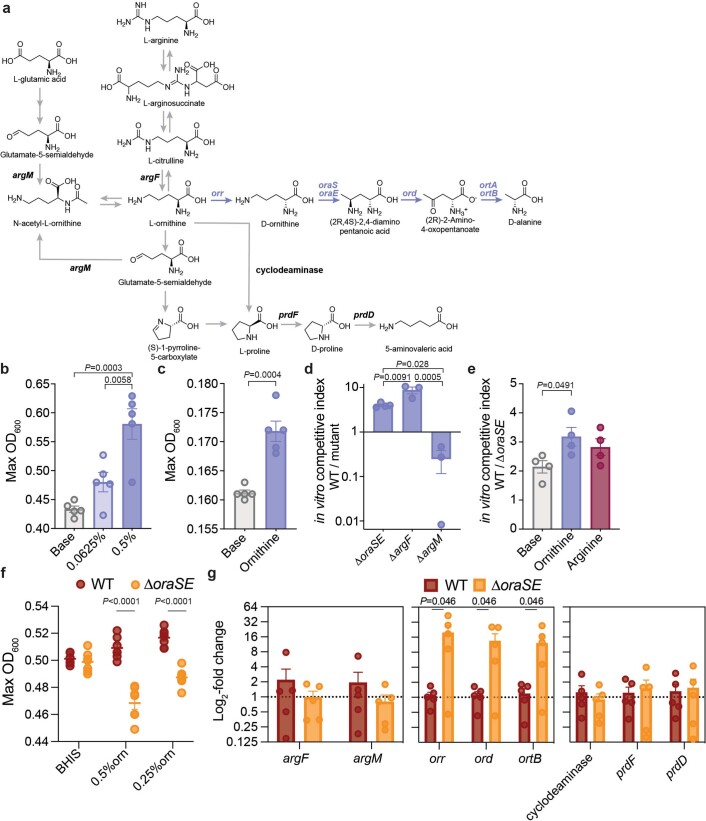
*Cd* ornithine degradation and biosynthesis pathways. **a**, Annotated biosynthetic and degradative pathways of L-ornithine in *Cd*. **b**, R20291 WT exhibits increased growth yield in rich media (0.5X BHIS) supplemented with different concentrations of L-ornithine (mean ± s.e.m., n = 5/group; one-way ANOVA F_(2,12)_=16.80 with Tukey’s post hoc comparisons). **c**, 630 WT exhibits increased growth yield in fully defined amino acid basal defined media (BDM) when supplemented with 8 mM L-ornithine (mean ± s.e.m., n = 5/group, unpaired two-tailed t-test). **d**, *In vitro* competition between 630 WT and mutants in L-ornithine degradation *(∆oraSE* n = 4) or biosynthesis *(∆argF*, n = 4; *∆argM*, n = 3). Strains were co-cultured for 24 hours in amino acid minimal media (CDDM) supplemented with additional 50 mM L-ornithine (mean ± s.e.m.; ANOVA with post hoc comparisons: F_(2,7)_=25.63,). A competitive index of 1 indicates equal abundance. **e**, *In vitro* competitive index between WT *Cd* and *∆oraSE* in BDM supplemented with 8 mM L-ornithine or 8 mM L-arginine (mean ± s.e.m., n = 4/group; ANOVA with Dunnett’s post hoc comparisons using base medium as the control group: F_(2,9)_=3.553). Strains were co-cultured for 48 hours. For all panels, * *P* < 0.05, ** *P* < 0.01, *** *P* < 0.001. **f**, WT *Cd* (n = 6) and *∆oraSE* (n = 7) grow equally well in rich media (0.5X BHIS). Supplementation with 0.5% or 0.25% (w/v) L-ornithine leads to increased growth yield in WT but not the ornithine aminomutase mutant (n = 7/group, mean ± s.e.m.). **g**, In gnotobiotic mice harboring a defined community of bacteria, the *∆oraSE* mutant exhibits increased expression of genes in the oxidative ornithine locus (middle panel) but not the reductive locus (right) nor biosynthesis of arginine (*argF*) or glutamine (*argM*). For **f** and **g**, multiple unpaired t-tests with Benjamini, Krieger and Yekutieli two-stage step-up FDR correction. Source data

Ornithine metabolism has not been investigated in *Cd* previously; based on homology in other members of the genus *Clostridium*, it is predicted that Stickland metabolism-based oxidative degradation of l-ornithine yields l-alanine, with the production of ATP via acetyl-CoA and ammonia (Fig. 2f)^28–31^. While the Stickland reaction typically occurs in amino acid pairs, other Clostridia can utilize ornithine as both an electron donor and acceptor. In addition to the oxidative catabolism of l-ornithine (Fig. 2f), *Cd* encodes a reductive pathway that generates 5-aminovalerate through proline (Extended Data Fig. 3a). We and others have demonstrated a role for proline reduction in *Cd* persistence in vivo^13,24^.

Addition of l-ornithine to medium increased growth yield of both *Cd* R20291 and 630 (Extended Data Fig. 3b,c). We constructed deletion mutants in ornithine oxidative degradation (*∆oraSE*) and two separate predicted ornithine biosynthetic pathways from arginine/citrulline (*∆argF*) or glutamine/glutamate (*∆argM*; Extended Data Fig. 3a). *∆oraSE* and *∆argF* suffered a competitive disadvantage after 24 h compared to WT *Cd* in amino acid minimal medium, but *∆argM*, involved in interconversion of glutamine and ornithine, does not (Extended Data Fig. 3d). These data suggest that ornithine oxidative degradation and biosynthesis from arginine/citrulline are more important than biosynthesis from glutamine for increasing culture density during growth on amino acids. Addition of ornithine increased the advantage of WT over *∆oraSE*, whereas arginine did not, indicating a potential fitness advantage results from ornithine taken up from the environment, rather than derived from *Cd*’s own metabolism (Extended Data Fig. 3e). In rich medium, WT and *∆oraSE* reached the same maximum optical density; addition of l-ornithine to rich medium increased growth of WT but not *∆oraSE* (Extended Data Fig. 3f). To determine whether the ornithine aminomutase deletion affected other ornithine metabolic pathways in vivo, we infected gnotobiotic mice harboring a defined consortium of bacteria with either WT or *∆oraSE* and isolated total RNA from cecal contents (Extended Data Fig. 3g). The mutant upregulated genes in the ornithine oxidative degradation locus (*orr*, *ord* and *ortB*), despite its inability to perform this metabolism. The ornithine reductive pathway (*cyclodeaminase*, *prdF* and *prdD*) as well as genes involved in ornithine interconversion with citrulline/arginine (*argF*) or glutamate/glutamine (*argM*) were unaffected.

**Fig. 3:**
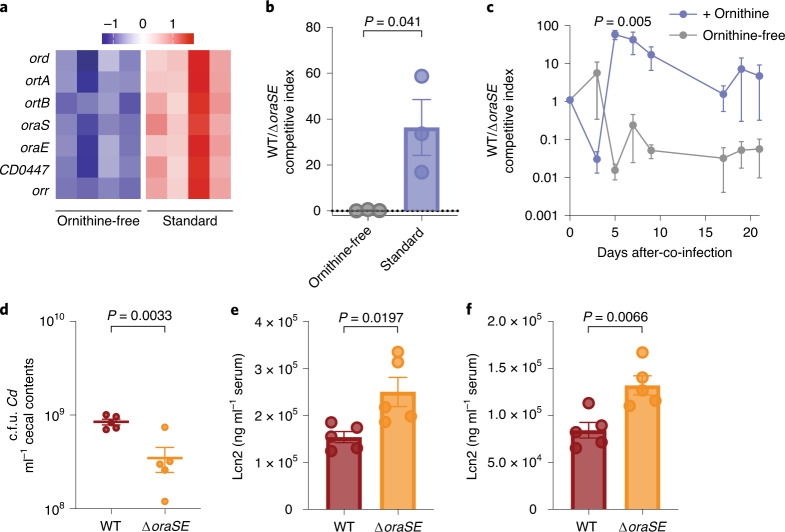
Metabolism of ornithine from diet confers competitive advantage to *Cd* and leads to reduced host inflammation. **a**, The ornithine oxidative degradation pathway is significantly enriched in mice monocolonized with WT *Cd* on standard mouse chow compared to mice monocolonized with WT *Cd* provided a fully defined diet lacking ornithine (row-normalized *z* score for microarray data from ref. ^32^; *n* = 4 mice per group). **b**, WT *Cd* has a competitive advantage in conventional mice over *∆oraSE* strain in standard diet background, but not in a fully defined diet devoid of ornithine (unpaired two-tailed Student’s *t*-test, *n* = 3 mice per group; mean ± s.e.m.). **c**, 1% ornithine supplementation (w/v) to conventional mice on a fully defined ornithine-free diet provides a competitive advantage to WT *Cd* (pairwise Student’s *t*-tests; mean ± s.e.m., *n* = 5 mice per group). **d**, WT *Cd* achieves a higher absolute abundance than ∆*oraSE* in cecal contents of gnotobiotic mice fed an ornithine-free diet supplemented with ornithine in drinking water. c.f.u., colony-forming unit. **e**,**f**, ∆*oraSE* infection leads to higher levels of lipocalin-2 in serum in gnotobiotic mice harboring a defined consortium of bacteria (**e**) or conventional mice fed standard diet (**f**). For **d**–**f** data were analyzed by unpaired two-tailed Student’s *t*-tests; mean ± s.e.m., *n* = 5 mice per group. Source data

To determine whether dietary ornithine availability influenced *Cd* metabolism, we compared transcriptional profiling^32^ of mice monocolonized with WT *Cd* fed standard rodent diet (which contains ornithine) or a fully defined diet lacking ornithine. Genes in the oxidative ornithine degradation locus were some of the most differentially expressed between dietary conditions: when dietary ornithine was present, genes involved in its oxidative metabolism were more highly expressed (Fig. 3a). To determine whether ornithine degradation conferred a fitness advantage in vivo, conventional microbiota-colonized mice were co-infected with equal amounts of WT and *∆oraSE Cd*. WT outcompeted the *∆oraSE* mutant in conventional mice fed standard diet containing ornithine, but not a fully defined diet devoid of ornithine (Fig. 3b). To test whether ornithine itself provided the competitive advantage to WT *Cd* over *∆oraSE*, conventional mice were fed a fully defined diet devoid of ornithine and co-infected with WT and *∆oraSE*. One group was administered 1% (w/v) l-ornithine in drinking water, which provided a competitive advantage to WT *Cd* over *∆oraSE*; WT did not outcompete the mutant in mice when dietary ornithine was lacking (Fig. 3c).

**Extended Data Fig. 4:**
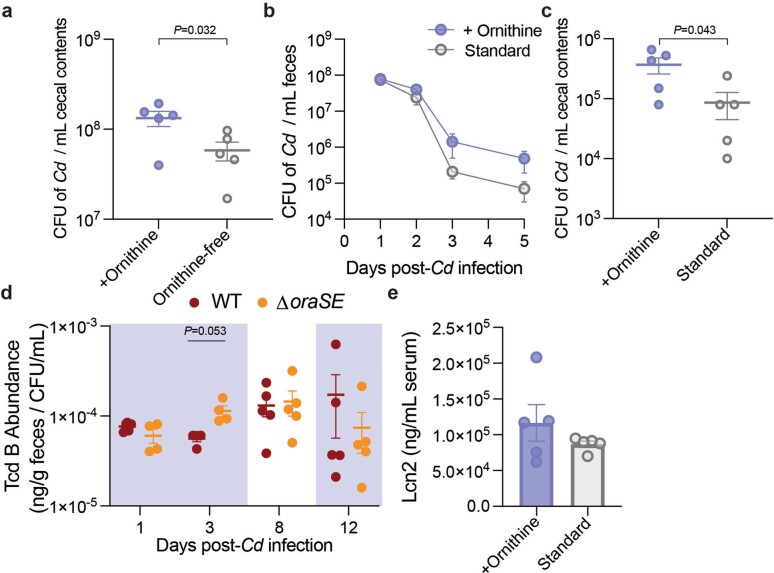
Dietary ornithine boosts *Cd* abundance in the mouse gut. **a**, Ornithine supplementation (1% w/v in drinking water) to conventional mice fed an ornithine-free diet co-infected with equal amounts WT and *∆oraSE* leads to higher overall *Cd* abundance in cecal contents (mean ± s.e.m., n = 5 mice/group; unpaired two-tailed t-test). WT *Cd* reaches higher abundance in feces (**b**, n = 5 mice/group, mean ± s.e.m.) and cecal contents (**c**, n = 5 mice/group, mean ± s.e.m.; unpaired two-tailed t-test) of conventional mice fed standard diet supplemented with 1% ornithine in drinking water. **d**, Dietary ornithine availability does not alter toxin production on a per-cell basis in mice harboring a defined consortium of bacteria infected with WT or *∆oraSE Cd* fed an ornithine-free diet (1% ornithine w/v administered in drinking water, denoted by shaded blue boxes; n = 5 mice/group, mean ± s.e.m.; multiple unpaired t-tests, FDR-adjusted with two-stage linear step-up). **e**, Exogenous ornithine provided in drinking water (1% w/v) to conventional mice fed standard diet does not influence serum lipocalin-2 levels (mean ± s.e.m., n = 5 mice/group). Source data

To determine whether dietary ornithine conferred a benefit in colonization levels, we infected gnotobiotic mice harboring a defined community with either WT or *∆oraSE*. WT achieved significantly higher abundance in cecal contents of mice fed an ornithine-free diet supplemented with ornithine in drinking water (Fig. 3d). In mice with a complex microbiota (conventional) fed an ornithine-free diet, ornithine supplementation in drinking water led to higher overall *Cd* abundance in cecal contents (Extended Data Fig. 4a). Finally, WT achieved higher absolute abundance in feces (Extended Data Fig. 4b) and cecal contents (Extended Data Fig. 4c) when ornithine was supplemented in drinking water to standard diet compared to standard diet alone.

We wanted to know whether *Cd* ornithine metabolism could affect severity of host inflammation. In both gnotobiotic (Fig. 3e) and conventional mice (Fig. 3f), ∆*oraSE* induced significantly higher levels of serum lipocalin-2, a readout of systemic host inflammation, than WT, despite lower overall abundance (Fig. 3d) and independent of significant differences in toxin production (Extended Data Fig. 4d). Dietary ornithine supplementation itself did not alter toxin production (Extended Data Fig. 4d) or serum lipocalin-2 (Extended Data Fig. 4e), suggesting that altered physiology or metabolism of the *∆oraSE* strain itself influences host inflammatory response and the ability to metabolize ornithine by WT *Cd* results in less inflammation.

**Extended Data Fig. 5:**
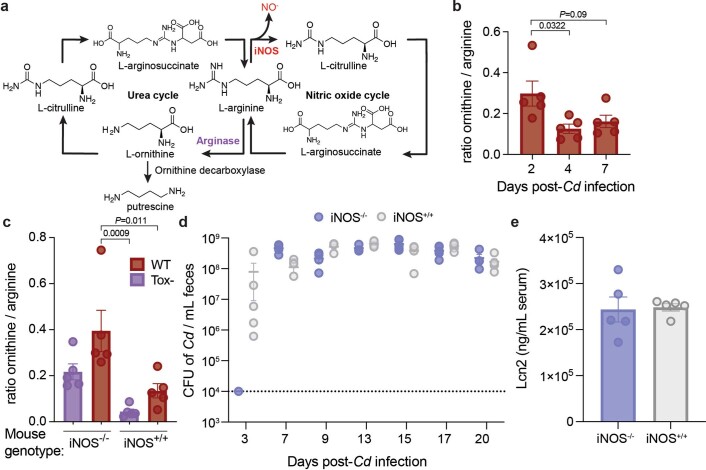
Host ornithine metabolism via inducible nitric oxide synthase. **a**, Intersecting host pathways act on arginine, producing nitric oxide (NO^−^) during an inflammatory response or ornithine via arginase during homeostatic or tissue-repair conditions. **b**, The ratio of ornithine to arginine in feces of conventional mice infected with WT *Cd* 630 (mean ± s.e.m., n = 5 mice/group; one-way ANOVA with Tukey’s post hoc comparisons: F_(2, 12)_=4.742). **c**, Fecal ratio of ornithine to arginine in *Cd*-infected (R20291 WT or R20291 Tox-) mice fed standard diet depends on host iNOS genotype (mean ± s.e.m., n = 5 mice/group; one-way ANOVA with Tukey’s post hoc comparisons: F_(3,16)_=8.614). Total *Cd* burden (**d**) and serum lipocalin-2 levels (**e**) in iNOS^−/−^ or iNOS^+/+^ mice fed a fully defined ornithine-free diet co-infected with WT and *∆oraSE*. For **d** and **e**, mean ± s.e.m. shown; n = 5 mice/group. Source data

In mammals, ornithine plays a role in immunometabolism; arginase breaks arginine down to ornithine, which is a precursor for the polyamines spermidine, spermine and putrescine (Extended Data Fig. 5a)^33–36^. Nitric oxide synthase also uses arginine as a substrate to generate NO^−^ during a reactive oxygen burst. Arginase activity has been described as the hallmark of a type-2-like inflammatory response as opposed to type 1, especially in macrophages^34^.

**Fig. 4:**
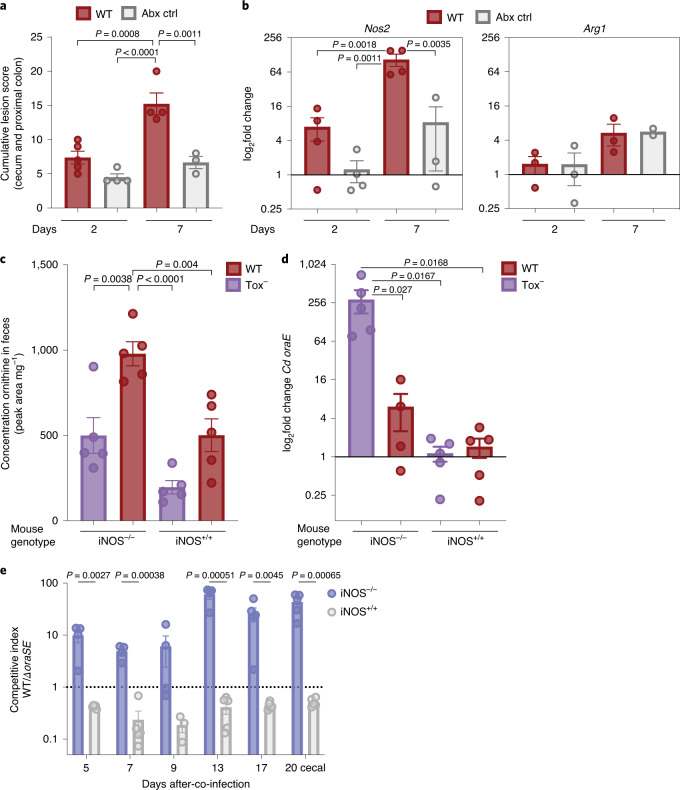
Ornithine produced via host iNOS-deficiency provides competitive advantage to *Cd* in the gut lumen. **a**, Blinded histopathology scoring of conventional mice fed standard diet infected with 630 WT 2 d and 7 d after infection (*n* = 5 mice per group) compared to antibiotic-treated controls (*n* = 4 mice, 2 d; *n* = 3 mice, 7 d; ANOVA F_(3,12)_ = 18.87 with Tukey’s post hoc comparisons; mean ± s.e.m.). **b**, iNOS expression in proximal colon tissue of mice infected with 630 WT is significantly higher 7 d after infection compared to 2 d and to uninfected, antibiotic-treated controls (WT day 7 significantly different from all other groups, ANOVA F_(3,11)_ = 12.97 with Tukey’s post hoc comparisons). Arginase-1 expression in the proximal colon tissue of mice is not significantly different between infected and uninfected mice. Expression levels are normalized to the antibiotic control group at day 2 (mean ± s.e.m., group sizes as in **a**). **c**, Genetic ablation of iNOS in mice fed standard diet leads to increased ornithine in feces during CDI (one-way ANOVA F_(3, 16)_ = 15.62 with Tukey’s post hoc comparisons; mean ± s.e.m., *n* = 5 mice per group). **d**, Increased expression of *Cd* ornithine aminomutase in iNOS^−/−^ mice infected with Tox^−^ *Cd* (expression levels normalized to WT in iNOS^+/+^ mice, one-way ANOVA F_(3,15)_ = 5.795 with Tukey’s multiple comparisons. Mean ± s.e.m., *n* = 5 mice per group except for WT-infected iNOS^−/−^ mice, where two outliers were removed with robust regression and outlier removal implemented in Prism 9 (ROUT, *Q* = 0.1)). **e**, WT *Cd* outcompetes *∆oraSE* in conventional iNOS^−/−^ but not iNOS^+/+^ mice fed a fully defined ornithine-free diet (multiple unpaired Student’s *t*-tests with Benjamini, Krieger and Yekutieli two-stage step-up FDR correction; mean ± s.e.m., *n* = 5 mice per group). Source data

We hypothesized that, in the absence of toxin-induced inflammation (Tox^−^), *Cd* may utilize host-derived ornithine. To determine whether host inducible nitric oxide synthase (iNOS) or arginase responds to CDI, we infected conventional mice with a standard antibiotic pretreatment (1 mg clindamycin per mouse by gavage) and subsequently infected with WT *Cd*. Compared to uninfected antibiotic-treated controls, mice infected with WT *Cd* suffered significantly higher tissue damage and inflammatory cell infiltrates quantified by blinded histopathological scoring (Fig. 4a). Host *Nos2*, but not *Arg1*, was upregulated concomitantly (Fig. 4b), suggesting that less arginine was available for ornithine biosynthesis under these conditions. Consistent with higher iNOS activity (increased NO^−^ generation), the ratio of ornithine to arginine in feces decreased from day 2 to 7 after infection (Extended Data Fig. 5b).

To increase the availability of arginine for ornithine biosynthesis via host arginase, we infected *Nos2* knockout mice. In the absence of host iNOS activity (iNOS^−/−^), the ratio of ornithine to arginine in feces was elevated during CDI compared to iNOS^*+/+*^ mice (Extended Data Fig. 5c), in alignment with increased iNOS activity limiting ornithine production during inflammation. The concentration of ornithine in feces is elevated in iNOS^−/−^ compared to iNOS^+/+^ mice (Fig. 4c) and *Cd* concurrently upregulated the d-ornithine aminomutase gene, *oraE* (Fig. 4d). In separate (WT or Tox^−^) infections, we could not rule out potential alterations in host immune response due to the *Nos2* genotype. To disentangle the specific metabolic interaction between host iNOS and the *Cd* oxidative ornithine pathway, we co-infected iNOS^−/−^ and iNOS^+/+^ mice on a fully defined ornithine-free diet with equal amounts of WT and ∆*oraSE Cd*. In alignment with our hypothesis that *Cd* utilizes host-derived ornithine, WT *Cd* outcompeted *∆oraSE* in iNOS^−/−^ but not iNOS^+/+^ mice (Fig. 4e). *Nos2* genotype did not affect *Cd* abundance after initial colonization (Extended Data Fig. 5d) nor systemic inflammation (Extended Data Fig. 5e). Thus, increasing host ornithine production via iNOS ablation led to increased expression of *Cd* oxidative ornithine utilization genes and provided a competitive advantage to WT *Cd*.

Here, we investigate *Cd* persistence in the presence of commensal community members in the absence of symptoms of disease. It has been demonstrated previously that enteric pathogens benefit from physiological changes imparted by their virulence factors^37–40^; however, the mechanisms by which *Cd* establishes asymptomatic carriage remain understudied.

We observed reduction of community amino acid metabolism in humanized mice that were susceptible to CDI and had elevated inflammation. Community pathways involved in ornithine, arginine and putrescine metabolism were reduced in *Cd*-susceptible mice before infection and absent after infection. Expression of oxidative ornithine metabolism unified *Cd* transcriptional expression in the absence of toxin production across two *Cd* strains and commensal communities. We previously reported that WT *Cd* exploits a metabolic product of the host inflammatory response^10^. Here, we demonstrate that it is possible for *Cd* to benefit from other host-derived compounds that are either non-inflammatory or are products of a type-2-like immune response. Our data are consistent with a recent study that conducted immunological transcriptional profiling of mouse cecal tissue in response to WT or non-toxigenic *Cd*^11^ and found increased *Nos2* expression but not *Arg1* in response to WT infection. Furthermore, *Cd* ornithine aminomutase was more highly expressed by a *tcdR* mutant (alternative sigma factor that induces toxin expression) compared to WT and expression of the ornithine utilization locus in WT *Cd* decreased when inflammation was higher in vivo^11^.

While Stickland metabolism-based ornithine metabolism has been investigated in other Clostridia, it had not been investigated in *Cd*, nor its implications investigated in vivo. We demonstrate that the ability to oxidatively degrade ornithine confers a competitive advantage due to the presence of dietary ornithine or host production of ornithine. This work highlights the metabolic cross-talk between host immunometabolism and *Cd* under homeostatic conditions in the gut. Understanding persistence of pathogens in the absence of disease is an important step toward preventative therapeutic strategies.

## Methods

### Mouse strains and husbandry

All animal experiments were performed in accordance with the Stanford Institutional Animal Care and Use Committee. For gnotobiotic mouse experiments, Swiss–Webster germ-free mice were maintained in gnotobiotic isolators. All animals were sex and age-matched and experiments were performed between 10 and 15 weeks of age. For conventional mouse experiments, Swiss–Webster Excluded Flora mice were used. For iNOS^−/−^ experiments, B6.129P2-Nos2^tm1Lau^/J mice from The Jackson Laboratory were ordered and C57BL/6J WT mice were used as a comparison. Mice were fed an autoclaved standard diet (Purina LabDiet 5010 for conventional, 5K67 for gnotobiotic) except in experiments where a fully defined diet lacking ornithine (BioServ Product, S6185) was used (noted in text and figure legends).

### Bacterial strains and growth conditions

*Cd*, *B.* *thetaiotaomicron* VPI-5482, *C.* *sporogenes* ATCC 35704, *C.* *scindens* ATCC 35704, *Clostridium* *bolteae* WAL-14578 and *Bifidobacterium* *longum* subspecies *infantis* ATCC 15697 strains were cultured anaerobically at 37 °C (85% N_2_, 10% CO_2_, 5% H_2_). *E.* *coli* K-12 MG1655 and *E.* *tarda* ATCC 23685 were grown aerobically at 37 °C in LB medium with 10 µg ml^−1^ vancomycin in plates. Solid medium used for *Cd* 630∆Erm and R20291 strains was CDMN (*Cd* agar base supplemented with 7% defibrinated horse blood, 0.5 mg ml^−1^ cysteine, 32 µg ml^−1^ moxalactam and 12 µg ml^−1^ norfloxacin) and CDDC (*Cd* agar base (Oxoid) supplemented with 7% defibrinated horse blood, 0.5 mg ml^−1^ cysteine, 250 µg ml^−1^ d-cycloserine and 16 µg ml^−1^ cefoxitin) for selective culture from mice and BHIS (Brain Heart Infusion Agar (Becton Dickinson) supplemented with 5 mg ml^−1^ yeast extract and 0.5 mg ml^−1^ cysteine) for routine culture from 25% (v/v) anaerobic glycerol stocks kept in glass crimp-top vials. Liquid medium used was BHIS (Brain Heart Infusion broth (BD) supplemented with yeast extract and 1 mg ml^−1^ cysteine. Fully defined minimal medium for growth curves and in vitro competition experiments was CDDM without glucose (*Cd* defined medium) and modified BDM (basal defined medium^41^). *B.* *thetaiotaomicron* was cultured on BHI Blood Agar plates (Brain Heart Infusion (BD) supplemented with 10% defibrinated horse blood and 200 µg ml^−1^ gentamycin; liquid medium used was BHIS for Bacteroides (Brain Heart Infusion agar (BD) supplemented with 5 µg ml^−1^ hemin and 2 µg ml^−1^ vitamin K1). TYG plates or broth were used for *C.* *sporogenes* culture (30 g l^−1^ tryptone, 20 g l^−1^ yeast extract, 1 g l^−1^ sodium thioglycolate; plates were supplemented with 125 µg ml^−1^ d-cyloserine, 38 µg ml^−1^ sulfamethoxazole and 2 µg ml^−1^ trimethoprim). *C.* *scindens* was cultured on CDMN plates and in TYG broth. *B.* *infantis* was cultured on de Man, Rogosa and Sharpe (Sigma-Aldrich) solid medium supplemented with 0.25% (w/v) l-cysteine and in TYG broth. *C.* *bolteae* was cultured on solid Reinforced Clostridial Agar (Sigma) and in Reinforced Clostridial Medium (Becton Dickinson).

### Gnotobiotic colonizations and pathogen infections

For experiments where transcriptional profiling and untargeted mass spectrometry was performed to compare WT and Tox^−^ infections versus uninfected control groups, germ-free mice were gavaged with 200 µl of a 1:1:1 mixture of the three community members (DC1, *B.* *thetaiotaomicron*, *C.* *sporogenes* and *E.* *coli* or DC2, *B.* *thetaiotaomicron*, *C.* *scindens* and *E.* *tarda*). Consortia were allowed to stabilize for 10–15 d in mice before introduction of *Cd* or were gavaged with anaerobic PBS for the uninfected control group. For experiments to determine the influence of dietary ornithine availability on longitudinal toxin production, germ-free mice were gavaged with 200 µl mixture of volumetrically equal parts *B.* *thetaiotaomicron*, *E.* *coli*, *C.* *bolteae* and *B.* *infantis*. The community stabilized for 13 d before *Cd* colonization.

In all gnotobiotic experiments, no antibiotic pretreatment was used before infection. Fourteen-hour *Cd* cultures grown in BHIS were fogged into isolator ports in 2-ml inner-threaded cryovials 2 h before infection such that mice were gavaged with 200 µl 16-h *Cd* culture grown in BHIS, corresponding to ~1 × 10^8^ c.f.u. ml^−1^.

For conventional mouse experiments, mice were gavaged with 1 mg clindamycin in 200 µl water 24 h before infection with 200 µl *Cd* grown in BHIS medium for 16 h. For co-infections, a 1:1 mixture of each *Cd* strain was used. If mice were fed an ornithine-free diet, diet was switched 24 h before antibiotic pretreatment (48 h before *Cd* infection). Bedding was replaced after diet switch, clindamycin gavage and after *Cd* infections.

### Generation of *Cd* mutants

The *Cd* 630∆Erm*∆pyrE∆oraSE*, *∆argF* and *∆argM* mutants were constructed via the *pyrE* allelic exchange system as previously described^42^. Briefly, regions 1 kb upstream and downstream of the targeted deletion region were amplified from purified WT genomic DNA (Supplementary Table 4) and inserted into the pMTL-YN3 vector backbone via Gibson Assembly, transformed into and propagated in *E.* *coli* TG1 before transformation into *E.* *coli* HB101/pRK24 conjugation-proficient cells. The pMTL-YN3 deletion plasmid was transferred to *Cd* 630∆Erm∆pyrE via spot-plate mating conjugation. During conjugation and subsequent selection steps, *Cd* and *E.* *coli* were cultured anaerobically with higher atmospheric H_2_ atmospheric levels: 85% N_2_, 5% CO_2_ and 10% H_2_. Plasmid integrants were selected for on BHIS medium supplemented with 15 µg ml^−1^ thiamphenicol, 50 µg ml^−1^ kanamycin, 16 µg ml^−1^ cefoxitin and 5 µg ml^−1^ uracil, and deletion mutants on CDDM supplemented with 5 µg ml^−1^ uracil and 2 mg ml^−1^ 5-fluoroorotic acid. Deletion loci were sequence-verified, after which the *pyrE* locus was restored to the *∆oraSE* mutant with a second round of mutagenesis using the pMTL-YN1C plasmid.

### Histopathology scoring

Tissue segments of approximately 1 cm in length were excised from the cecal blind tip and most proximal colonic section of mice with a clean razor blade, placed in plastic cassettes, fixed in formalin for 24–48 h, then transferred to 70% ethanol for long-term storage. Tissues were embedded in paraffin, sectioned and stained with hematoxylin and eosin. Tissues (cecal blind tip and proximal colon) were given scores of 0–3 in each of the following parameters: inflammatory cell infiltration, mucosal hyperplasia, vascular congestion, epithelial disruption and submucosal edema. The cumulative lesion score is the sum of each score in these independent categories. Scoring was performed blinded.

### 16S sequencing and analysis

Total DNA was extracted from frozen fecal samples using the DNeasy PowerSoil HTP 96 kit (QIAGEN). Barcoded primers were used to amplify the V3–V4 region of the 16S rRNA gene using 515f and 806r primers. The Ultra Clean 96 well PCR Clean-Up kit (QIAGEN) was used to clean the amplicons before quantification of DNA yield (Quant-iT), library pooling and quality control with BioAnalyzer. The 250–300-bp paired-end reads were generated on Illumina MiSeq2500. Demultiplexing was performed with Qiime 1.9 (ref. ^43^) and reads were assigned to a custom 16S rRNA reference database of the defined communities using pick_closed_reference_otus.py.

### RNA-seq and analysis

#### RNA isolation

RNA from cecal contents was isolated using phenol:chloroform extraction followed by cleanup with the RNEasy Mini kit (QIAGEN) or using the RNA Power Microbiome kit (QIAGEN) per the manufacturer’s instructions.

#### Library prep and sequencing

Ribosomal RNA was depleted from total RNA isolated from cecal contents using the Illumina Ribo-Zero Gold Epidemiology rRNA Removal kit. Depletion of rRNA and RNA quality was verified with Agilent Bioanalyzer Prokaryote Total RNA Pico before moving forward with library preparation using the Illumina TruSeq mRNA Stranded HT Library Prep kit. Sequencing for in vivo RNA-seq was performed on an Illumina HiSeq4000 instrument with 100-bp paired-end reads.

#### Analysis

Raw, paired reads were imported into CLC Genomics Workbench v.11 with a maximum distance of 1,000 bp. In vivo reads were trimmed with a quality limit of 0.05, an ambiguous limit of 2, based on automatic read-through adaptor trimming, with a minimum number of nucleotides of 50. Broken pairs and discarded sequences were not saved. Reads were mapped to the following reference genomes: *Cd* 630 genome (RefSeq accession NC_009089.1), *Cd* R20291 (RefSeq, FN545816.1), *B.* *thetaiotaomicron* VPI-5482 (RefSeq, NC_004663.1), *E.* *coli* str. K-12 substr. MG1655 (RefSeq, NC_000913.3), *C.* *scindens* ATCC 35704 (RefSeq, NZ_CP036170.1), *E.* *tarda* ATCC 23685 (RefSeq, NZ_ADGK00000000.1) and *C.* *sporogenes* ATCC 15579 (RefSeq, NZ_ABKW00000000.2). Standard parameters were used; paired reads were mapped to the gene track with a mismatch cost of 2, insertion cost of 3, deletion cost of 3 and length and similarity fractions of 0.8. Strand specificity was used; paired distances were auto-detected and reads that mapped in pairs were counted as one hit. A maximum of ten hits per read was allowed. Total read counts for each gene were exported and remaining ribosomal RNA reads were manually removed before normalization and differential expression analysis in R with *DESeq2* (ref. ^44^).

For differential expression analysis, comparable experimental conditions (same defined community, same sequencing run) were combined into one DESeq object to be fitted with a negative binomial GLM. Low-count reads were filtered at a count threshold of eight for 630 and five for R20291. Pairwise contrasts were specified between the experimental groups to define significance with the Wald test.

### RT–qPCR

RNA from frozen host proximal colon tissue was extracted with the RNEasy Mini kit; 25–30 mg tissue was bead-beat with acid-washed glass beads in 600 µl RLT + ßME for 5 min at 4 °C before proceeding with the kit protocol. Complementary DNA was generated using random primers and Superscript III Reverse Transcriptase per the manufacturer’s instructions; RNase OUT was included. Quantitative PCR (qPCR) was performed with SYBR Brilliant III on an Agilent MX3000P or CFX Connect Real-Time System in 20-µl volumes with 0.3 µl reference dye added. Changes to expression were calculated by normalizing Ct value to *Gapdh* (host tissue) or *rpoA* (*Cd* expression) with the Δ-Δ Ct method.

### Competition assays

In vivo and in vitro competition between WT and mutant strains was assessed using qPCR. Primer pairs (Supplementary Table 4) targeting unique loci within the WT *oraE*, *argF* or *argM* genes (WT) or surrounding their respective deletions were validated with serial dilutions of purified genomic DNA. Efficiency values for each primer pair were calculated as 10^(1/-slope)^ of log_10_(DNA input) against the Ct value. For in vivo competition, total DNA was extracted from frozen fecal samples of conventional mice co-infected with WT and *∆oraSE Cd* using the DNeasy PowerLyzer PowerSoil kit (QIAGEN). For in vitro competitions, 5-ml cultures were co-inoculated with 1:100 diluted combined (1:200 dilution each) overnight culture of WT and a mutant strain. After 24 h growth, cells were pelleted and total DNA was extracted by modification of existing protocols^45^ as follows: one wash with 1× TE, incubation in 50 µl 50 mg ml^–1^ lysozyme and 200 µl genomic DNA solution for 30 min at 37 °C, 100 µl 20% Sarkosyl and 15 µl 10 mg ml^–1^ RNase A for 30 min at 37 °C, 7.5 µl 20 mg ml^–1^ proteinase K and 200 µl buffer AL (QIAGEN) for 30 min at 56 °C, addition of 200 µl 100% ethanol before proceeding with the DNeasy Blood and Tissue kit (QIAGEN). qPCR reactions were performed as described above. The competitive index of each genotype was calculated as 2^−Ct^ WT primer pair / 2^−Ct^ mutant primer pair. A competitive index of 1 indicates that each strain is at equal abundance.

### Toxin B quantification

The tgcBIOMICS Toxin B ELISA kit (TGC-E002-1) was used to measure *Cd* TcdB concentration in mouse feces or cecal contents with anti-tcdB-HRP. The 25–50 mg frozen fecal samples were thawed, weighed and resuspended thoroughly in 450 µl dilution buffer. Resuspended fecal samples were allowed to sediment and 100 µl supernatant was used in the assay, proceeding via the manufacturer’s instructions. Toxin levels were quantified (ng toxin per g feces) using a 1:2 dilution series standard curve in duplicate (40 ng ml^–1^ to 0.31 ng ml^–1^) and normalized to the absolute abundance of *Cd* levels (c.f.u. ml^–1^) from the same mouse, as previously described^10^.

### Lipocalin-2 quantification

Mouse serum lipocalin-2 levels were measured using the Mouse Lipocalin-2/NGAL DuoSet ELISA kit (R&D Biosystems). Mouse serum previously snap-frozen in liquid nitrogen and stored at −80 °C was thawed on wet ice, mixed and diluted 1:250 and 1:1,000 in 1× PBS containing protease inhibitor (Roche cOmplete Mini Protease Inhibitor Cocktail) before proceeding via the manufacturer’s instructions. Quantification was performed with a ten-point standard curve in triplicate.

### Mass spectrometry assays

#### Untargeted quantification of cecal metabolites

Germ-free Swiss–Webster mice harboring a defined community of bacteria (*B.* *thetaiotaomicron*, *C.* *sporogenes* and *E.* *coli*) were infected with *Cd* WT R20291, R20291 Tox^−^ or gavaged with anaerobic PBS as a control. After 5 d of infection, cecal contents were collected and flash-frozen in liquid nitrogen. Cecal contents were weighed to ~10 mg and shipped on dry ice to the West Coast Metabolomics Center at University of California, Davis. For the WT-infected group, two fecal samples were included due to limited volume of cecal contents in the hypervirulent infection, which were also used for RNA-seq. Then, 4 ± 0.3 mg samples were extracted, derivatized and analyzed by gas chromatography time of flight mass spectrometry (GC–TOF–MS) as described previously^46^. Reported data (Supplementary Table 1) represent peak heights for each ion normalized to the sum of the peak heights (mTIC) for all named metabolites within a treatment group. Identified metabolites (*n* = 220) were included in downstream analysis. Peak areas were log-transformed and statistical differences between groups was performed with Metaboanalyst^47^.

#### Targeted quantification of amino acids

We modified an existing protocol^48^ to quantify amino acids from complex biological matrices. Feces were thawed and weighed before metabolite extraction. Fecal samples were subsequently resuspended in 1:1 LC–MS-grade acetonitrile:water, beat with acid-washed glass beads for 5 min at 4 °C, incubated for 5 min at room temperature and centrifuged for 5 min. Then, 50 µl supernatant was mixed with 25 µl 0.1 M sodium tetraborate and 50 µl dansyl chloride (50 mM in 100% acetonitrile). Samples were incubated for 30 min at room temperature in the dark, mixed at the 15-min interval. Then, 50 µl 0.5% formic acid was added to stop the derivatization reaction and samples were subsequently filtered through a 0.45-µm low-binding filter plate (Millipore). A 5-µl sample was injected onto a 10-cm C18 column (Acquity) with a flow rate of 0.5 ml min^–1^ in 0.1% formic acid in water (solvent A) and 0.1% formic acid in acetonitrile (solvent B). Solvent A started at 95% and B at 5%; from 0–10.25 min, A decreased to 40%; 10.25–12.5 min, A decreased to 5%; 12.5–15 min A held at 5%; 15–15.1 min A increased to 95% and held until the end of the run at 18 min. Column temperature was held at 40 °C throughout. Metabolites were detected with an Agilent 6460 Triple Quad mass spectrometer in positive ionization mode using 300.11 (l-ornithine), 303.63 (d_7_-l-ornithine) and 408.17 (l-arginine) precursor ions, with 171 as the product. A 100 [ms] dwell time was used for l-arginine and 150 [ms] for l-ornithine; 120 [V] fragmentor voltage for l-arginine and 100 [V] for l-ornithine; 40 [V] collision energy for l-arginine and 10 [V] for l-ornithine. Peaks were identified and integrated with Agilent MassHunter Workstation Software Quantitative Analysis. External standard curves of l-ornithine were generated and d_7_-l-ornithine was used as an internal standard during protocol development.

### Analysis of previous datasets

Human microbial communities classified as dysbiotic or healthy-like are described in our previous work^13^. In brief, 16S rRNA sequencing was performed on samples provided by healthy human donors (*n* = 118) and samples from patients presenting with diarrhea (*n* = 115). Microbial composition separated patients into two clusters: dysbiotic (lower α- and β-diversity) versus healthy. Partitioning around medoids, the gap statistic and average silhouette width clustering analyses were used to categorize samples; all healthy donors fell into one category (healthy-like), whereas the samples provided by patients with diarrhea were separated into healthy-like and dysbiotic. A subset (six each from the healthy-like and dysbiotic groups) of these human samples were chosen for mouse humanization and subsequent challenge with *Cd*. RNA-seq was performed on fecal pellets before and after *Cd* infection; gene and pathway expression profiles were obtained with HUMAnN2 v.0.5.0. RNA-seq results were normalized to shallow metagenomic reads; pathway differential expression analysis was performed with DESeq2 v.1.8.2 and LEfSe.

Published microarray data^32^ are available through the Gene Expression Omnibus under the accession number GSE60751. Analysis comparing the two *Cd*-monocolonized groups (standard diet versus polysaccharide-deficient diet) was performed in GEO2R with standard analysis parameters.

### Reporting Summary

Further information on research design is available in the Nature Research Reporting Summary linked to this article.

## Supplementary information

Reporting Summary

Supplementary Table 1 Untargeted GC–TOF metabolomics of gnotobiotic mice harboring a defined community infected with WT R20291, R20291 Tox^−^ or uninfected.

Supplementary Table 2 Differential expression analysis of 630 WT versus 630 Tox^−^ in the presence of DC1 and 2; *DESeq2* Wald test, Benjamini–Hochberg FDR correction.

Supplementary Table 3 Differential expression analysis of R20291 WT versus R20291 Tox^−^ in the presence of DC1; *DESeq2* Wald test, Benjamini–Hochberg FDR correction.

Supplementary Table 4 List of primers used in this study.

## Data Availability

Raw RNA-seq data are available at NCBI SRA with accession no. PRJNA687238; untargeted GC–TOF metabolomics data are available at Metabolomics Workbench (https://www.metabolomicsworkbench.org/) under accession no. ST001650.

## Source data

Source Data Fig. 1 Source data for Fig. 1.
Source Data Fig. 2 Source data for Fig. 2.
Source Data Fig. 3 Source data for Fig. 3.
Source Data Fig. 4 Source data for Fig. 4.
Source Data Extended Data Fig. 1 Source data for Extended Data Fig. 1.
Source Data Extended Data Fig. 2 Source data for Extended Data Fig. 2.
Source Data Extended Data Fig. 3 Source data for Extended Data Fig. 3.
Source Data Extended Data Fig. 4 Source data for Extended Data Fig. 4.
Source Data Extended Data Fig. 5 Source data for Extended Data Fig. 5.

## Code availability

Code is available at https://github.com/kpruss/Cdiff-ornithine. Source data are provided with this paper.

## References

[1] Lessa FC (2015). Burden of *Clostridium difficile* infection in the United States. N. Engl. J. Med..

[2] Crobach MJT (2018). Understanding *Clostridium difficile* colonization. Clin. Microbiol. Rev..

[3] Schäffler H, Breitrück A (2018). *Clostridium difficile* - from colonization to infection. Front. Microbiol..

[4] Abt MC, McKenney PT, Pamer EG (2016). *Clostridium difficile* colitis: pathogenesis and host defence. Nat. Publ. Gr..

[5] Rousseau C (2012). *Clostridium difficile* carriage in healthy infants in the community: a potential reservoir for pathogenic strains. Clin. Infect. Dis..

[6] Kyne L, Warny M, Qamar A, Kelly CP (2000). Asymptomatic carriage of *Clostridium difficile* and serum levels of IgG antibody against toxin A. N. Engl. J. Med..

[7] Nissle K, Kopf D, Rösler A (2016). Asymptomatic and yet *C. difficile*-toxin positive? Prevalence and risk factors of carriers of toxigenic *Clostridium difficile* among geriatric in-patients. BMC Geriatr..

[8] Kong LY (2015). Predictors of asymptomatic *Clostridium difficile* colonization on hospital admission. Am. J. Infect. Control.

[9] Furuya-Kanamori L (2015). Asymptomatic *Clostridium difficile* colonization: epidemiology and clinical implications. BMC Infect. Dis..

[10] Pruss KM, Sonnenburg JL (2021). *C. difficile* exploits a host metabolite produced during toxin-mediated infection. Nature.

[11] Fletcher JR (2021). *Clostridioides difficile* exploits toxin-mediated inflammation to alter the host nutritional landscape and exclude competitors from the gut microbiota. Nat. Commun..

[12] Rojo, D. et al. *Clostridium difficile* heterogeneously impacts intestinal community architecture but drives stable metabolome responses. *ISME J*. 10.1038/ismej.2015.32 (2015). 10.1038/ismej.2015.32 PMC4579473 25756679

[13] Battaglioli EJ (2018). *Clostridioides difficile* uses amino acids associated with gut microbial dysbiosis in a subset of patients with diarrhea. Sci. Transl. Med..

[14] Fritz JH (2013). Arginine cools the inflamed gut. Infect. Immun..

[15] Cynober L (1994). Can arginine and omithine support gut functions?. Gut.

[16] Rooks MG, Garrett WS (2016). Gut microbiota, metabolites and host immunity. Nat. Rev. Immunol..

[17] Robinson JI (2019). Metabolomic networks connect host-microbiome processes to human *Clostridioides difficile* infections. J. Clin. Invest..

[18] Lei XH, Bochner BR (2013). Using Phenotype MicroArrays to determine culture conditions that induce or repress toxin production by *Clostridium difficile* and other microorganisms. PLoS ONE.

[19] Kuehne SA (2010). The role of toxin A and toxin B in *Clostridium difficile* infection. Nature.

[20] Merrigan M (2010). Human hypervirulent *Clostridium difficile* strains exhibit increased sporulation as well as robust toxin production. J. Bacteriol..

[21] Warny M (2005). Toxin production by an emerging strain of associated with outbreaks of severe disease in North America and Europe. Lancet.

[22] Kuehne SA (2014). Importance of toxin A, toxin B, and CDT in virulence of an epidemic *Clostridium difficile* strain. J. Infect. Dis..

[23] Liu, Y. et al. Electron transfer complexes in the gut dictate high abundance circulating metabolites. Preprint at *bioRxiv*10.1101/2019.12.11.873224 (2019).

[24] Bouillaut L, Self WT, Sonenshein AL (2013). Proline-dependent regulation of *Clostridium difficile* stickland metabolism. J. Bacteriol..

[25] Anderson CJ, Clark DE, Adli M, Kendall MM (2015). Ethanolamine signaling promotes *Salmonella* niche recognition and adaptation during infection. PLoS Pathog..

[26] Thiennimitr P (2011). Intestinal inflammation allows *Salmonella* to use ethanolamine to compete with the microbiota. Proc. Natl Acad. Sci. USA.

[27] Kendall MM, Gruber CC, Parker CT, Sperandio V (2012). Ethanolamine controls expression of genes encoding components involved in interkingdom signaling and virulence in enterohemorrhagic *Escherichia coli* O157:H7. MBio.

[28] Mitruka BM, Costilow RN (1967). Arginine and ornithine catabolism by *Clostridium botulinum*. J. Bacteriol..

[29] Dyer JK, Costilow RN (1968). Fermentation of ornithine by *Clostridium sticklandii*. J. Bacteriol..

[30] Tsuda Y, Friedmann C (1970). Ornithine metabolism by *Clostridium**sticklandii*. J. Biol. Chem..

[31] Fonknechten N (2009). A conserved gene cluster rules anaerobic oxidative degradation of L-ornithine. J. Bacteriol..

[32] Ferreyra JA (2014). Gut microbiota-produced succinate promotes *C. difficile* infection after antibiotic treatment or motility disturbance. Cell Host Microbe.

[33] Galván-peña S, O’Neill LAJ (2014). Metabolism reprograming in macrophage polarization. Front. Immunol..

[34] Rath M, Müller I, Kropf P, Closs EI, Munder M (2014). Metabolism via arginase or nitric oxide synthase: two competing arginine pathways in macrophages. Front. Immunol..

[35] Kim, Y.-G. et al. Gut dysbiosis promotes M2 macrophage polarization and allergic airway inflammation via fungi-induced PGE2. *Cell Host Microbe*10.1016/j.chom.2013.12.010 (2014). 10.1016/j.chom.2013.12.010 PMC3957200 24439901

[36] Das P, Lahiri A, Lahiri A, Chakravortty D (2010). Modulation of the arginase pathway in the context of microbial pathogenesis: a metabolic enzyme moonlighting as an immune modulator. PLoS Pathog..

[37] Winter SE (2010). Gut inflammation provides a respiratory electron acceptor for *Salmonella*. Nature.

[38] Lopez CA (2016). Virulence factors enhance *Citrobacter rodentium* expansion through aerobic respiration. Science.

[39] Faber F (2016). Host-mediated sugar oxidation promotes post-antibiotic pathogen expansion. Nature.

[40] Rivera-Chávez F, Mekalanos JJ (2019). Cholera toxin promotes pathogen acquisition of host-derived nutrients. Nature.

[41] Karasawa T, Ikoma S, Yamakawa K (1995). A defined growth medium for *C. difficile*. Microbiology.

[42] Ng YK (2013). Expanding the repertoire of gene tools for precise manipulation of the *Clostridium difficile* genome: allelic exchange using pyrE alleles. PLoS ONE.

[43] Caporaso JG (2010). QIIME allows analysis of high-throughput community sequencing data. Nat. Methods.

[44] Love MI, Huber W, Anders S (2014). Moderated estimation of fold change and dispersion for RNA-seq data with DESeq2. Genome Biol..

[45] Bouillaut L, McBride SM, Sorg JA (2011). Genetic manipulation of *Clostridium difficile*. Curr. Protoc. Microbiol..

[46] Fiehn O (2017). Metabolomics by gas chromatography-mass spectrometry: the combination of targeted and untargeted profiling. Curr. Protoc. Mol. Biol..

[47] Pang Z, Chong J, Li S, Xia J (2020). Metaboanalystr 3.0: toward an optimized workflow for global metabolomics. Metabolites.

[48] Wu M (2013). Opiate-induced changes in brain adenosine levels and narcotic drug responses. Neuroscience.

