# Supplementary material for: Oxidative ornithine metabolism supports non-inflammatory C. difficile colonization
Source: Nat Metab. 2022 Jan 6;4(1):19–28. doi: 10.1038/s42255-021-00506-4 (PMC8803604; doi:10.1038/s42255-021-00506-4)
Supplement: Supplementary file 1 — Reporting Summary [file 42255_2021_506_MOESM1_ESM.pdf]

nature research

Corresponding author(s): Justin L. Sonnenburg

Last updated by author(s): 10/27/21

Reporting Summary

Nature Research wishes to improve the reproducibility of the work that we publish. This form provides structure for consistency and transparency in reporting. For further information on Nature Research policies, see our Editorial Policies and the Editorial Policy Checklist.

Statistics

For all statistical analyses, confirm that the following items are present in the figure legend, table legend, main text, or Methods section.

n/a	Confirmed	
☐	☒	The exact sample size (n) for each experimental group/condition, given as a discrete number and unit of measurement
☐	☒	A statement on whether measurements were taken from distinct samples or whether the same sample was measured repeatedly
☐	☒	The statistical test(s) used AND whether they are one- or two-sided<br>*Only common tests should be described solely by name; describe more complex techniques in the Methods section.*
☐	☒	A description of all covariates tested
☐	☒	A description of any assumptions or corrections, such as tests of normality and adjustment for multiple comparisons
☐	☒	A full description of the statistical parameters including central tendency (e.g. means) or other basic estimates (e.g. regression coefficient) AND variation (e.g. standard deviation) or associated estimates of uncertainty (e.g. confidence intervals)
☐	☒	For null hypothesis testing, the test statistic (e.g. F, t, r) with confidence intervals, effect sizes, degrees of freedom and P value noted<br>*Give P values as exact values whenever suitable.*
☒	☐	For Bayesian analysis, information on the choice of priors and Markov chain Monte Carlo settings
☒	☐	For hierarchical and complex designs, identification of the appropriate level for tests and full reporting of outcomes
☒	☐	Estimates of effect sizes (e.g. Cohen's d, Pearson's r), indicating how they were calculated

Our web collection on statistics for biologists contains articles on many of the points above.

Software and code

Policy information about availability of computer code

Data collection	RNA-seq was performed on Illumina HiSeq4000. Agilent MassHunter Data Acquisition software was used for targeted LC/MS amino acid data collection and Agilent MassHunter Quantitative Analysis software was used for peak integration. BioTek Gen5 software was used to acquire optical density readings for bacterial growth curves and ELISAs.
Data analysis	CLC Genomics Workbench version 11 was used for RNA-seq read-mapping. R package DESeq2 v1.32.0 was used to perform RNA-seq differential expression analysis. Metaboanalyst v4.0 was used to analyze untargeted GC-TOF metabolomics data. Qiime v1.9.1 was used for 16S amplicon analysis. For community-level pathway differential expression analysis: gene and pathway expression profiles were obtained with HUMAnN2 v0.5.0 and pathway differential expression analysis was performed with LEfSe and DESeq2 v1.8.2. Prism (Graphpad) version 9 was used for all other statistical analyses and visualizations.<br><br>Code is available on github, at https://github.com/kpruss/Cdiff-ornithine.

For manuscripts utilizing custom algorithms or software that are central to the research but not yet described in published literature, software must be made available to editors and reviewers. We strongly encourage code deposition in a community repository (e.g. GitHub). See the Nature Research guidelines for submitting code & software for further information.

Data

Policy information about availability of data

All manuscripts must include a data availability statement. This statement should provide the following information, where applicable:

- Accession codes, unique identifiers, or web links for publicly available datasets
- A list of figures that have associated raw data
- A description of any restrictions on data availability

Raw RNA-seq data has been deposited to NCBI SRA with the accession number PRJNA687238; untargeted GC-TOF metabolomics data has been deposited to Metabolomics Workbench (https://www.metabolomicsworkbench.org/) under the accession number ST001650.

Field-specific reporting

Please select the one below that is the best fit for your research. If you are not sure, read the appropriate sections before making your selection.

☒ Life sciences ☐ Behavioural & social sciences ☐ Ecological, evolutionary & environmental sciences

For a reference copy of the document with all sections, see nature.com/documents/nr-reporting-summary-flat.pdf

Life sciences study design

All studies must disclose on these points even when the disclosure is negative.

Sample size	No statistical methods were used to pre-determine sample size. Sample sizes were chosen based on animal litter numbers and controlling for sex and age within experiments.
Data exclusions	For panel 4d, two outliers were removed with robust regression and outlier removal implemented in Prism 9 (ROUT, Q=0.1)).
Replication	C. difficile 630 transcriptional profiling was repeated twice (in the presence of 2 distinct defined consortia of bacteria) and the findings were reproducible. Minimal media growth curves for C. difficile 630 and the ornithine aminomutase mutant were repeated at least twice and the findings were reproducible.
Randomization	Fixed mouse tissue samples were randomized for histopathological scoring. For all animal experiments, littermates were grouped by a researcher unaware of experimental design. Grouped littermates were assigned to treatment arms randomly prior to beginning experimentation.
Blinding	Researchers were not blinded to groups during data collection except for in histopathological scoring, where the scorer was fully blinded as well as to the numbers and sizes of groups.

Reporting for specific materials, systems and methods

We require information from authors about some types of materials, experimental systems and methods used in many studies. Here, indicate whether each material, system or method listed is relevant to your study. If you are not sure if a list item applies to your research, read the appropriate section before selecting a response.

Materials & experimental systems

n/a	Involved in the study	
☒	☐	Antibodies
☒	☐	Eukaryotic cell lines
☒	☐	Palaeontology and archaeology
☐	☒	Animals and other organisms
☒	☐	Human research participants
☒	☐	Clinical data
☒	☐	Dual use research of concern

Methods

n/a	Involved in the study	
☒	☐	ChIP-seq
☒	☐	Flow cytometry
☒	☐	MRI-based neuroimaging

Animals and other organisms

Policy information about studies involving animals; ARRIVE guidelines recommended for reporting animal research

Laboratory animals	For gnotobiotic mouse experiments, Swiss-Webster germ-free mice were maintained in gnotobiotic isolators. All animals were sex and age-matched and experiments were performed between 10-15 weeks of age. For conventional mouse experiments, Swiss-Webster Excluded Flora mice were used. For iNOS-/- experiments, B6.129P2-Nos2tm1Lau/J mice from The Jackson Laboratory were ordered and C57BL/6J wild-type mice were used as a comparison.

Wild animals	The study did not involve wild animals.
Field-collected samples	The study did not involve samples collected from the field.
Ethics oversight	All animal experiments were performed in accordance with the Stanford Institutional Animal Care and Use Committee.

Note that full information on the approval of the study protocol must also be provided in the manuscript.
